# Severe Hyponatremia Following Nitrofurantoin Therapy: A Case of Suspected Syndrome of Inappropriate Antidiuretic Hormone Secretion (SIADH)

**DOI:** 10.7759/cureus.105374

**Published:** 2026-03-17

**Authors:** Sikandar Afzal, Alison Ryan, Melinda Rou Yen Heng, Fergal Coleman, Muhammad Sajjad Sadiq

**Affiliations:** 1 Endocrinology, Mayo University Hospital, Castlebar, IRL; 2 General Internal Medicine, Mayo University Hospital, Castlebar, IRL; 3 Endocrinology and Diabetes, Mayo University Hospital, Castlebar, IRL

**Keywords:** adverse drug reaction, antibiotic complications, drug-induced siadh, electrolyte imbalance, fluid restriction, hyponatremia, hypotonic hyponatremia, nitrofurantoin, siadh

## Abstract

Nitrofurantoin is a widely prescribed antibiotic for the treatment of uncomplicated urinary tract infections, but it is an uncommon cause of the syndrome of inappropriate antidiuretic hormone secretion (SIADH). We report the case of a previously healthy 68-year-old woman who developed acute, severe hypotonic hyponatremia due to SIADH shortly after initiating nitrofurantoin therapy for a presumed urinary tract infection. She presented to the emergency department with persistent nausea, vomiting, dizziness, and generalized weakness two days after starting nitrofurantoin. Laboratory investigations revealed profound hyponatremia with inappropriately concentrated urine, normal renal, thyroid, and adrenal function, and no evidence of infection or malignancy, consistent with SIADH. Nitrofurantoin was promptly discontinued, and the patient was managed in the intensive care unit with strict fluid restriction and close biochemical monitoring. Her serum sodium levels gradually normalized over several days, with complete resolution of symptoms. This case highlights nitrofurantoin as a rare but clinically significant trigger of SIADH, particularly in older adults. Early recognition, prompt withdrawal of the offending medication, and appropriate management are essential to prevent potentially life-threatening complications. Increased clinical awareness of antibiotic-induced SIADH may facilitate earlier diagnosis and safer prescribing practices.

## Introduction

Hyponatremia is the most common electrolyte abnormality encountered in clinical practice, affecting up to 15-30% of hospitalized patients and is associated with increased morbidity, mortality, and length of hospital stay [1]. Among its various aetiologies, the syndrome of inappropriate antidiuretic hormone secretion (SIADH) represents a leading cause of euvolemic hypotonic hyponatremia. SIADH is characterized by inappropriately concentrated urine and impaired free-water excretion in the presence of normal circulating volume, renal function, adrenal reserve, and thyroid status [2].

Numerous conditions have been implicated in the pathogenesis of SIADH, including pulmonary disorders, central nervous system disease, malignancy, postoperative states, and medications. Drug-induced SIADH is an important yet frequently under-recognized cause, particularly in older adults who are more susceptible to the effects of medications on renal water handling [3]. Commonly implicated drug classes include antidepressants, anticonvulsants, antipsychotics, chemotherapeutic agents, and certain antibiotics; however, nitrofurantoin has only rarely been reported as a causative agent, with evidence largely limited to isolated case reports.

Nitrofurantoin is widely prescribed as first-line therapy for uncomplicated urinary tract infections due to its favourable antimicrobial spectrum and generally good safety profile [4]. Although gastrointestinal upset and hypersensitivity reactions are well-recognized adverse effects, serious electrolyte disturbances are seldom reported. Recent literature has highlighted isolated cases of antibiotic-associated SIADH culminating in severe neurological complications such as generalized tonic-clonic seizures [5], underscoring the potential for life-threatening outcomes when this condition is overlooked.

We present a case of severe hypotonic hyponatremia consistent with SIADH occurring shortly after nitrofurantoin exposure, highlighting the importance of careful medication review and early recognition of rare but clinically significant adverse drug reactions.

## Case presentation

A 68-year-old woman with no significant past medical history initially presented to her general practitioner with a one-week history of dysuria and urinary frequency. She was prescribed cefalexin 500 mg twice daily, which she took for three days without symptomatic improvement. She subsequently sought further medical review and was commenced on nitrofurantoin 100 mg twice daily. Two days after starting nitrofurantoin, she developed persistent nausea, vomiting, dizziness, and generalized weakness, prompting attendance at the emergency department (ED).

On arrival, she was alert and oriented with a Glasgow Coma Scale (GCS) score of 15/15 and was hemodynamically stable. She appeared clinically euvolemic, with no signs of dehydration (no dry mucous membranes or reduced skin turgor) and no evidence of fluid overload (no peripheral oedema or raised jugular venous pressure). Cardiovascular, respiratory, and abdominal examinations were unremarkable.

A detailed neurological examination was performed. Cranial nerve assessment was normal, with no facial asymmetry, dysarthria, dysphagia, or visual field deficits. There was no spontaneous or gaze-evoked nystagmus. Pupils were equal and reactive to light. Motor examination demonstrated normal tone, power (5/5 in all limbs), and symmetrical reflexes. Sensory examination was intact to light touch and pinprick in all four limbs. Cerebellar testing revealed normal finger-nose coordination and heel-shin testing, with no dysmetria or ataxia. Gait assessment was limited due to generalized weakness but showed no focal neurological deficit. There were no signs of meningeal irritation.

Initial laboratory investigations demonstrated severe hyponatremia (serum sodium 110 mmol/L) with low serum osmolality (228 mOsm/kg). Potassium, renal, liver, and thyroid function tests were within normal limits. Early-morning cortisol was 550 nmol/L, excluding adrenal insufficiency. Admission laboratory investigations are summarized in Table 1, and urinary studies are presented in Table 2.

**Table 1:** Admission laboratory investigations Laboratory investigations on admission demonstrated severe hyponatremia and serum osmolality (228 mOsm/kg), while other measured parameters, including renal, liver, thyroid, and inflammatory markers, were within normal limits. eGFR: estimated glomerular filtration rate, ALP: alkaline phosphatase, ALT: alanine aminotransferase, GGT: gamma-glutamyl transferase, NT-proBNP: N-terminal prohormone of brain natriuretic peptide, TSH: thyroid-stimulating hormone

Parameters	Result	Reference range
Sodium (mmol/l)	110	136 to 146
Potassium (mmol/l)	3.7	3.5 to 5.1
Glucose (mmol/l)	4.8	4.1 to 7.8
Creatinine (umol/l)	46	49 to 90
Urea (mmol/l)	2.2	2.8 to 7.2
eGFR (ml/min)	>90	>90
Calcium (mmol/l)	2.28	2.2 to 2.65
Serum osmolality ( mOsm/kg)	228	275-295
Total protein (g/l)	63	66 to 83
Albumin (g/l)	35	35 to 52
Total bilirubin (umol/l)	21	0 to 21
ALP (u/l)	70	30 to 120
ALT (u/l)	65	0 to 35
GGT (u/l)	29	0 to 38
Magnesium (mmol/l)	0.69	0.77 to 1.03
C-Reactive Protein (mg/l)	1.6	0 to 5
NT-proBNP	254	<400
TSH (mIU/L)	1.80	0.38 to 5.33
Vitamin B12 (pg/ml)	496	130 to 705
Folate (ng/ml)	7.4	2.6 to 20.6
Early morning cortisol	550nmol/L	>140nmol/L
Haemoglobin	14	12 to 15
White cell count	7000	4 to 10
Platelets	250000	150 to 410

**Table 2:** Admission urinary studies Urine results meet the diagnosis criteria of syndrome of inappropriate antidiuretic hormone secretion (SIADH).

Parameters	Result	Reference range
Urine urea (mmol/L)	155.6	N/A
Urine creatinine (mmol/L)	6.3	N/A
Urine sodium (mmol/L)	41.2	N/A
Urine potassium (mmol/L)	47.0	N/A
Urine osmolality (mOsm/kg)	339	(50 to 1500)
Urine glucose (mmol/L)	0.42	(0.1 to 0.8)

Urinary analysis demonstrated inappropriately concentrated urine (osmolality 339 mOsm/kg) with elevated urine sodium (41.2 mmol/L) in the absence of diuretic use. In the context of clinical euvolemia with normal adrenal, thyroid, and renal function, these findings were consistent with SIADH.

**Figure 1:**
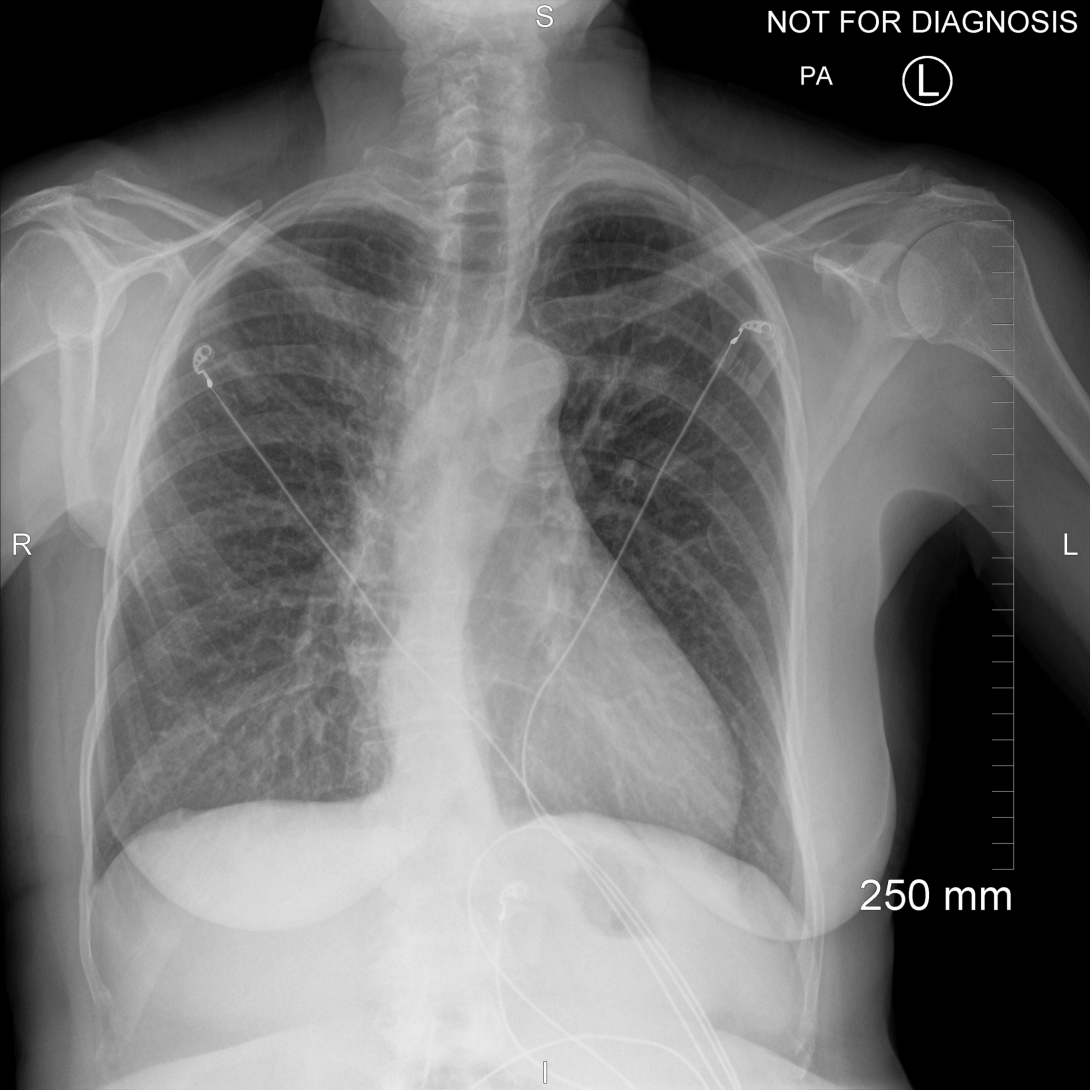
Chest radiograph demonstrated no acute abnormality

**Figure 2:**
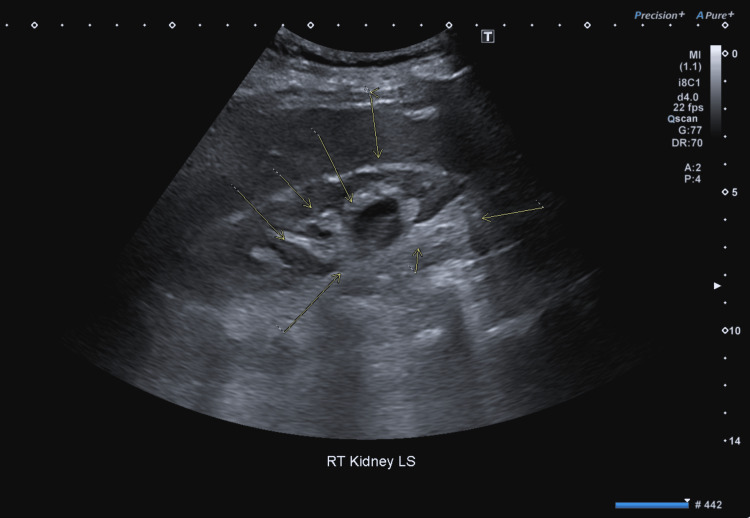
Renal ultrasound showing unilateral scarring Renal ultrasound demonstrated no acute abnormality. Incidental unilateral cortical scarring was noted.

Chest radiograph showed no acute abnormality (Figure 1). Urine microscopy was negative for infection. Renal ultrasound demonstrated unilateral renal scarring (Figure 2), likely representing chronic changes and not contributing to the acute presentation. Computed tomography of the thorax, abdomen, and pelvis was unremarkable. The diagnostic criteria for SIADH and corresponding patient findings are summarized in Table 3.

**Table 3:** Diagnostic criteria for SIADH and corresponding patient findings SIADH: syndrome of inappropriate antidiuretic hormone secretion; TSH: thyroid-stimulating hormone; eGFR: estimated glomerular filtration rate

SIADH criteria	Findings
Hypotonic hyponatremia	Serum sodium 110 mmol/L with low serum osmolality (228 mOsm/kg)
Inappropriately concentrated urine	Urine osmolality (339 mOsm/kg)
Elevated urine sodium concentration in the absence of diuretic use.	Urine sodium concentration (41.2 mmol/L)
Clinical euvolemia	No signs of dehydration or fluid overload on examination
Normal thyroid and adrenal function	Normal TSH and cortisol
Normal renal function	eGFR > 90 mL/min/1.73 m^2^

The patient was transferred to the intensive care unit (ICU) for controlled correction of serum sodium. Nitrofurantoin was discontinued, and fluid restriction to 750 mL/day was initiated with close monitoring of serum sodium every four to six hours. The rate of correction did not exceed 8 mmol/L in any 24-hour period. Over the subsequent 48-72 hours, serum sodium increased gradually and safely, with complete resolution of symptoms. Serial serum sodium measurements demonstrating the rate of correction are summarized in Table 4.

**Table 4:** Serial serum sodium measurements Serial serum sodium measurements demonstrating gradual correction of severe hyponatremia following discontinuation of nitrofurantoin and initiation of fluid restriction. The rate of correction did not exceed 8 mmol/L per 24 hours, remaining within recommended safety thresholds to minimize the risk of osmotic demyelination syndrome.

Hospital day	Serum sodium (mmol/L)
Day 1 (Admission)	110
Day 2	118
Day 3	124
Day 4	129
Day 5	135

Outcome and follow-up

The patient demonstrated steady clinical and biochemical improvement following discontinuation of nitrofurantoin and implementation of fluid restriction to 750 mL/day. She remained clinically stable throughout her admission, with no neurological or hemodynamic abnormalities observed. Serum sodium levels increased gradually and returned to the normal range without complications. Serial laboratory results during admission are summarized in Table 5, with renal, thyroid, adrenal, and inflammatory markers remaining within normal limits throughout her hospital stay. Once electrolyte stability was achieved, the patient was discharged in good clinical condition. At a two-week outpatient follow-up, she remained asymptomatic, and her serum sodium was stable at 134 mmol/L. She was advised to avoid nitrofurantoin in the future due to the risk of recurrent SIADH.

**Table 5:** Serial laboratory investigations at discharge and two-week follow-up Serial laboratory results demonstrate normalization of serum sodium following treatment. eGFR: estimated glomerular filtration rate, ALP: alkaline phosphatase, ALT: alanine aminotransferase, GGT: gamma-glutamyl transferase

Parameters	Result (on discharge)	Results (on follow-up)	Reference range
Sodium (mmol/l)	135	134	136 to 146
Potassium (mmol/l)	4.1	4.2	3.5 to 5.1
Glucose (mmol/l)	4.5	5.6	4.1 to 7.8
Creatinine (umol/l)	70	66	49 to 90
Urea (mmol/l)	4.1	5.3	2.8 to 7.2
eGFR (ml/min)	77	82	>90
Calcium (mmol/l)	2.26	N/A	2.2 to 2.65
Albumin (g/l)	36	40	35 to 52
Total bilirubin (umol/l)	13	10	0 to 21
ALP (u/l)	81	78	30 to 120
ALT (u/l)	54	14	0 to 35
GGT (u/l)	31	20	0 to 38
Magnesium (mmol/l)	0.87	N/A	0.77 to 1.03
C-reactive protein (mg/l)	12.9	1.9	0 to 5

## Discussion

The present case highlights a rare but clinically significant adverse effect of nitrofurantoin-induced SIADH leading to severe hyponatremia. European clinical practice guidelines define SIADH as euvolemic hypotonic hyponatremia with inappropriately concentrated urine and elevated urinary sodium excretion in the absence of adrenal insufficiency, hypothyroidism, renal dysfunction, or ongoing diuretic therapy [6]. Alternative causes of hyponatremia were systematically excluded. Computed tomography of the thorax, abdomen, and pelvis was unremarkable, with no radiological evidence of malignancy. Infectious causes were unlikely given the absence of systemic or respiratory symptoms on admission and a normal C-reactive protein level. There was no clinical or radiographic evidence of pulmonary disease. Endocrine causes were excluded by normal thyroid function tests and an adequate early-morning cortisol level. Renal function was preserved, and the patient was not receiving diuretics or other regular medications known to impair water homeostasis. Collectively, these findings support a drug-induced etiology.

Drug-induced hyponatremia is well recognized as a major contributor to inpatient electrolyte disturbances. A comprehensive review demonstrated that medications are responsible for a substantial proportion of SIADH cases, particularly in hospitalized and elderly populations [3,4,7]. Antibiotics represent an underappreciated subgroup of causative agents compared with psychotropic or antiepileptic medications. Proposed mechanisms include increased central release of antidiuretic hormone (ADH), potentiation of renal tubular responsiveness to ADH, and direct renal effects impairing free-water excretion [7]. Recognition of these mechanisms is crucial, as early identification and withdrawal of the causative drug often leads to rapid biochemical and clinical improvement.

Although nitrofurantoin is widely prescribed due to its favourable safety profile, its potential to cause serious systemic adverse reactions warrants vigilance. Pulmonary toxicity remains the most frequently reported severe complication, mediated through immune and oxidative pathways [8]. Electrolyte disturbances, by contrast, are exceedingly rare, with very few documented cases in the literature. A notable prior report described life-threatening generalized seizures associated with nitrofurantoin-induced hyponatremia, emphasizing that neurological manifestations may be the presenting feature and that outcomes can be severe when diagnosis is delayed [5]. Similarly, antibiotic-induced SIADH has been described with other agents, including cefoperazone/sulbactam, which produced clinically significant hyponatremia in a documented case report [7]. These reports reinforce the concept that antimicrobial therapy, though often overlooked as a causative factor, can precipitate dangerous disturbances in sodium homeostasis.

Management of drug-induced SIADH hinges on prompt cessation of the offending medication alongside standard supportive measures, most commonly fluid restriction, as recommended in established guidelines [9]. In severe or symptomatic cases, hypertonic saline therapy may be warranted with careful monitoring to avoid overly rapid correction and the risk of osmotic demyelination [10]. In the majority of reported drug-related cases, including nitrofurantoin-associated events, withdrawal of the antimicrobial agent results in gradual normalization of serum sodium and complete resolution of symptoms [7,10]. Our patient followed a similarly favourable course, further reinforcing the causal relationship and the effectiveness of conservative management when initiated promptly.

This case expands the limited body of evidence linking nitrofurantoin to SIADH and underscores the importance of clinician awareness of antimicrobial-associated electrolyte disturbances. Given the widespread prescription of nitrofurantoin for urinary tract infections, particularly among older patients who are at heightened risk of drug-induced hyponatremia, sodium monitoring and careful medication review should be considered in patients presenting with unexplained neurological or gastrointestinal symptoms during treatment. Early recognition remains the key determinant of patient outcome.

## Conclusions

Nitrofurantoin-associated SIADH appears to be an exceptionally rare but clinically significant adverse drug reaction. This case highlights the importance of maintaining a high index of suspicion for medication-related hyponatremia, particularly in older adults or those recently exposed to antibiotics. In our patient, the temporal association between nitrofurantoin initiation, the development of severe hypotonic hyponatremia, and subsequent biochemical improvement following drug discontinuation supports a possible relationship. Early recognition, prompt withdrawal of the suspected offending agent, and careful correction of serum sodium are essential to prevent neurological complications. Clinicians should consider monitoring electrolytes in high-risk patients receiving nitrofurantoin and include SIADH in the differential diagnosis when patients present with unexplained hyponatremia shortly after starting the medication. As evidence currently remains limited to isolated case reports, further reporting of similar cases may help clarify this potential association and improve clinical awareness.

## References

[1] Upadhyay A, Jaber BL, Madias NE (2006). Incidence and prevalence of hyponatremia. Am J Med.

[2] Verbalis JG, Goldsmith SR, Greenberg A, Korzelius C, Schrier RW, Sterns RH, Thompson CJ (2013). Diagnosis, evaluation, and treatment of hyponatremia: expert panel recommendations. Am J Med.

[3] Liamis G, Filippatos TD, Elisaf MS (2016). Thiazide-associated hyponatremia in the elderly: what the clinician needs to know. J Geriatr Cardiol.

[4] Muller AE, Verhaegh EM, Harbarth S, Mouton JW, Huttner A (2017). Nitrofurantoin's efficacy and safety as prophylaxis for urinary tract infections: a systematic review of the literature and meta-analysis of controlled trials. Clin Microbiol Infect.

[5] Sanni MO, Rajkanna J, Sagi SV, Oyibo SO (2023). Syndrome of inappropriate antidiuretic hormone secretion (SIADH) complicated by generalized tonic-clonic seizures after a course of antibiotics: a case report. Cureus.

[6] Spasovski G, Vanholder R, Allolio B (2014). Clinical practice guideline on diagnosis and treatment of hyponatraemia. Eur J Endocrinol.

[7] Mitra S, Basu S (2006). Cefoperazone/sulbactam induced hyponatremia. Indian J Med Sci.

[8] Kaye AD, Shah SS, LaHaye L (2025). Nitrofurantoin-induced pulmonary toxicity: mechanisms, diagnosis, and management. Toxics.

[9] Ayus JC, Moritz ML, Fuentes NA (2025). Correction rates and clinical outcomes in hospitalized adults with severe hyponatremia: a systematic review and meta-analysis. JAMA Intern Med.

[10] Squadrito FJ, Del Portal D (2023). Nitrofurantoin. StatPearls [Internet].

